# The Development and Consequences of Red Blood Cell Alloimmunization

**DOI:** 10.1146/annurev-pathol-042320-110411

**Published:** 2022-11-09

**Authors:** Connie M. Arthur, Sean R. Stowell

**Affiliations:** Joint Program in Transfusion Medicine, Department of Pathology, Brigham and Women’s Hospital, Harvard Medical School, Boston, Massachusetts

**Keywords:** red blood cell, alloimmunization, hemolysis, sickle cell disease, transfusion medicine

## Abstract

While red blood cell (RBC) transfusion is the most common medical intervention in hospitalized patients, as with any therapeutic, it is not without risk. Allogeneic RBC exposure can result in recipient alloimmunization, which can limit the availability of compatible RBCs for future transfusions and increase the risk of transfusion complications. Despite these challenges and the discovery of RBC alloantigens more than a century ago, relatively little has historically been known regarding the immune factors that regulate RBC alloantibody formation. Through recent epidemiological approaches, in vitro–based translational studies, and newly developed preclinical models, the processes that govern RBC alloimmunization have emerged as more complex and intriguing than previously appreciated. Although common alloimmunization mechanisms exist, distinct immune pathways can be engaged, depending on the target alloantigen involved. Despite this complexity, key themes are beginning to emerge that may provide promising approaches to not only actively prevent but also possibly alleviate the most severe complications of RBC alloimmunization.

## INTRODUCTION

Blood transfusion is the most common medical intervention in hospitalized patients. However, despite the routine nature of allogeneic blood transfusion today, the history of blood transfusion started with highly unpredictable outcomes. The earliest attempts at transfusion were actual xenotransfusions, and while several of these transfusions appeared to be successful, not all were beneficial. One patient who received calf’s blood was recorded as having severe back pain and urine as black “as if it had been mixed with soot of chimneys” (1, p. 621). Further transfusion attempts led to his death. This is the first recorded case of immune-mediated destruction of transfused blood that resulted in a fatal hemolytic transfusion reaction (HTR) and illustrates the potential consequences of immune incompatibility following xeno- or allogeneic transfusion (2). Blood transfusion remained controversial due to its unpredictable nature, with a random mix of successful or fatal outcomes. The etiology of HTRs and the underlying mechanisms that led to distinct transfusion outcomes remained enigmatic until molecular differences between and within species were discovered by Karl Landsteiner in 1900. Through the discovery of distinct alloantibody reactivity between individuals, features of red blood cells (RBCs) responsible for immune-mediated destruction of allogeneic transfused blood that resulted in HTRs were determined (3). These studies resulted in the discovery of ABO(H) blood group antigens and corresponding anti-ABO(H) alloantibodies, which not only provided a critical framework for modern-day compatibility testing prior to transfusion but also became the first polymorphisms described within the human population (4, 5).

**Figure 1:**
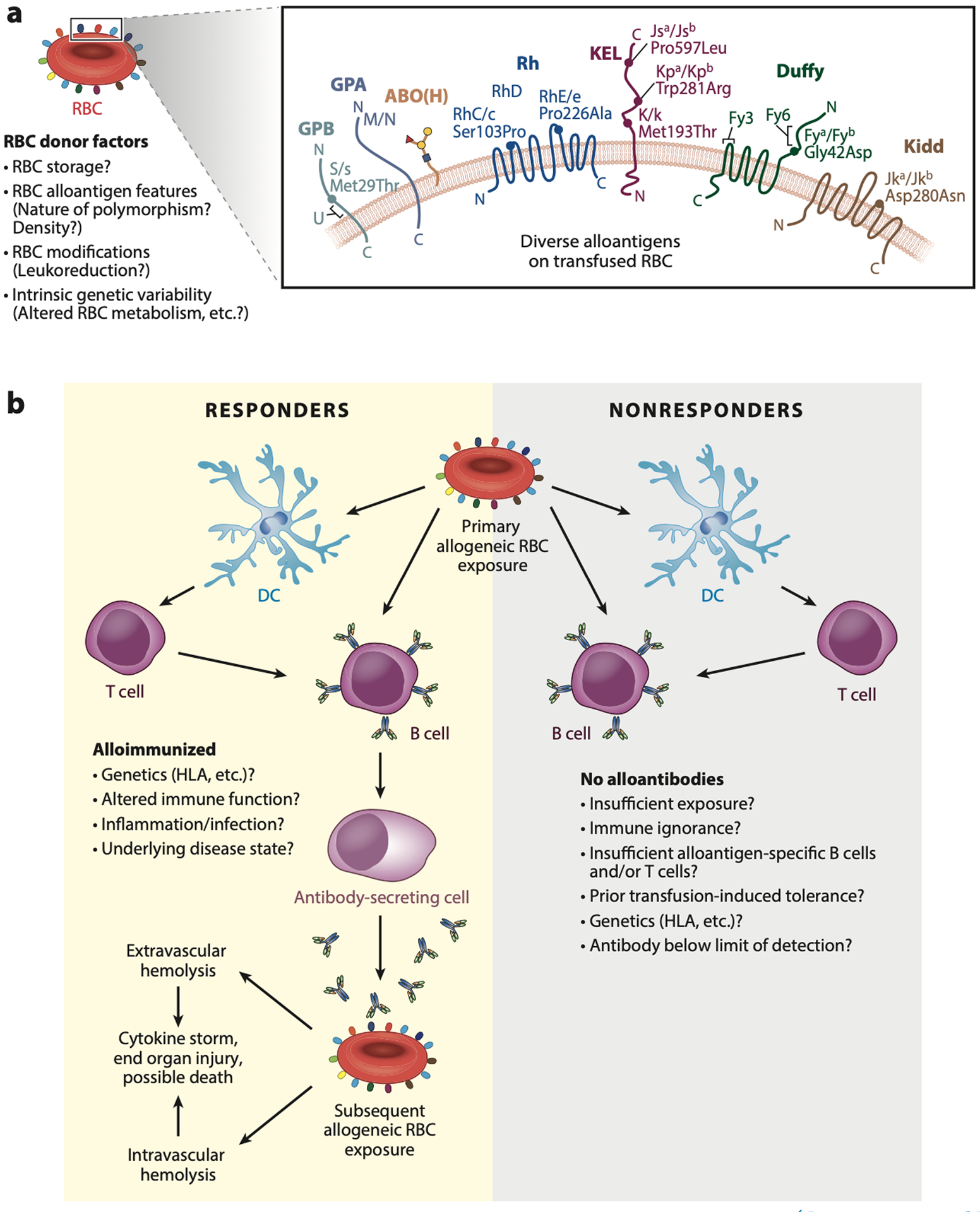
Red blood cell (RBC) alloimmunization. (*a*) More than 300 different alloantigens on RBCs have been described. In contrast to the spontaneous formation of anti-ABO(H) alloantibodies, individuals can develop additional alloantibodies as a result of direct exposure to RBC alloantigens (due to the lack of immunological tolerance toward the alloantigens not expressed by the transfusion recipient). RBC alloantigens can differ in density, chemical composition, and overall function. Some alloantigens reflect carbohydrate modifications [ABO(H)], while others represent the presence or absence of an entire protein (RhD). Most are due to a single amino acid substitution as shown for S/s, RhC/c, RhE/e, KEL (Js^b^/Js^a^, Kp^a^/Kp^b^, k/K), Duffy (Fy^a^/Fy^b^), and Kidd (Jk^a^/Jk^b^); some reflect changes in several amino acids as illustrated by M/N (M = Ser, Ser, Thr, Thr, Gly; N = Leu, Ser, Thr, Thr, Glu). (*b*) The general concept of the RBC alloimmune response suggests that RBC alloimmunization results from the uptake of transfused RBCs by antigen-presenting cells, such as dendritic cells (DCs), allowing the processing and presentation of alloantigen peptides in the context of major histocompatibility antigens to CD4 T cells. B cells also engage allogeneic RBCs and, with the help of CD4 T cells, can be driven to become antibody secreting cells (plasma cells) that produce IgG anti-RBC alloantibodies. However, despite many transfusion exposures, some patients, referred to as nonresponders, never generate detectable alloantibodies even though they are negative for a variety of RBC alloantigens to which they could, in theory, generate alloantibodies. In contrast, patients who have made alloantibodies following transfusion are called responders. The underlying basis for why responders generate alloantibodies and nonresponders do not has been the subject of many studies, where a variety of possibilities have emerged. It is certainly possible that nonresponders could generate alloantibodies under certain conditions, and therefore a hard line between responders and nonresponders may not exist. Instead, responder status may more likely reflect a continuum that is influenced by recipient genetics such as human leukocyte antigens (HLAs), donor unit variability, and the status of the immune system at the time of transfusion; ultimately, these factors may all dictate the likelihood that alloantibodies form following allogeneic RBC exposure.

As the practice of transfusion evolved, additional alloantibody reactivity toward RBCs distinct from ABO(H) alloantigens emerged (2, 6). Many of these findings predated the discovery of DNA as the hereditary material and provided the earliest evidence of multiple polymorphisms within patients (2). The first clinically relevant non-ABO(H) RBC alloantibodies were discovered in a mother who experienced an HTR after transfusion of ABO(H) compatible blood donated by the father of their child, following a hemorrhagic complication she experienced secondary to the birth of their stillborn child (6). Subsequent studies demonstrated that antibodies directed against Rhesus RBCs likewise reacted with the father’s but not the mother’s RBCs, resulting in the discovery of the Rhesus D (RhD) alloantigen (2). Maternal alloantibodies against the RhD alloantigen can cross the placenta and induce hemolysis of the developing fetus’s RhD-positive RBCs; this fetal RBC hemolysis was likely responsible for the demise of the stillborn child, a process now referred to as hemolytic disease of the fetus and newborn (HDFN). Additional incompatibilities detected following pregnancy or transfusion, distinct from ABO(H) and RhD, have also been described (2). Collectively, studies over decades of transfusion and pregnancy evaluation have revealed an entire array of RBC polymorphisms, now including more than 300 distinct RBC alloantigens (2,7). RBC polymorphism targets of alloantibodies are collectively referred to as blood group antigens, but these antigens can vary considerably in composition and structure, from the presence or absence of entire protein gene products to single amino acid variation (Figure 1). Alloantibodies that form against many of these alloantigens can cause HDFN or HTRs; as a result, specific procedures have been developed to prevent transfusion of incompatible blood products. When a type and screen is performed on a patient to provide compatible RBCs, the blood type refers to the individual’s ABO and RhD blood group status, while the screen is designed to detect any alloantibodies directed against non-ABO(H) blood group alloantigens.

Unlike anti-ABO(H) alloantibodies, which form spontaneously within the first few months of life (4), as noted for anti-RhD, alloantibodies against most other RBC polymorphisms are induced as a direct result of RBC alloantigen exposure following transfusion or pregnancy (6). In transfusion-dependent patients, the accumulation of alloantibodies against distinct alloantigens and/or the presence of alloantibodies against highly prevalent alloantigens can make it difficult to procure compatible RBCs that are negative for the corresponding alloantigen targets (7). As a result, these alloantibodies can directly prolong life-threatening anemia, put patients at risk for transfusion-related hemolytic complications, and place a developing fetus at risk for HDFN. Despite the long-standing knowledge of RBC alloimmunization and the challenge alloantibodies can present for patient care, RBC alloimmunization has been challenging to study. However, recent clinical, translational, and preclinical studies have begun to unveil the underlying mechanisms whereby alloantibodies form and the consequences of immune incompatibility following transfusion. These data provide promising new approaches that may be employed to reduce RBC alloimmunization and its attendant consequences. As RBC alloimmunization during pregnancy and the development of HDFN has been excellently reviewed elsewhere (8), in this review we focus on the development and consequences of alloimmunization following RBC transfusion.

## THE EPIDEMIOLOGY OF RBC ALLOIMMUNIZATION

While virtually all blood group O individuals spontaneously generate anti-A and anti-B alloantibodies, RBC-induced alloimmunization following transfusion or pregnancy is not a uniform outcome of RBC alloantigen exposure. Indeed, some patients, referred to as nonresponders, fail to generate alloantibodies despite many allogeneic RBC transfusions (Figure 1). In contrast, other patients, referred to as responders, have generated alloantibodies following a prior RBC alloantigen encounter. However, even among responders, RBC alloimmunization is an infrequent event. On average, RBC alloimmunization occurs approximately 0.4% of the time on a per unit basis (7). It is therefore often unclear if nonresponders are incapable of generating an immune response against allogeneic RBCs or if they simply haven’t experienced adequate exposure. It is also possible that some individuals may generate alloantibodies following RBC transfusion but that these alloantibodies are undetected. This can reflect alloantibody evanescence prior to the next alloantibody screen and/or inadequate sensitivity of commonly employed assays (most clinical assays are designed to detect incompatibility prior to RBC transfusion that could result in an HTR and are not equipped with the sensitivity of more commonly employed antibody-detection assays, such as flow cytometry or the enzyme-linked immunosorbent assay, to determine whether low-level alloantibodies may be present) (9–11). Irrespective of the reason a patient may or may not possess detectable alloantibodies following allogeneic RBC transfusion, the ability to directly predict responder versus nonresponder status a priori holds significant clinical value, as it would allow alloantigen matching protocols and possible immune inhibiting approaches to be directed toward individuals most at risk for developing alloantibodies following transfusion (12, 13). However, what is more likely than strict nonresponder or responder status is that a combination of genetic and environmental factors makes an individual more prone to becoming alloimmunized following a particular transfusion event and that responders and nonresponders therefore reflect a continuum (Figure 1).

### Recipient Factors

Among genetic factors that can influence RBC alloimmunization, immune-related polymorphisms, most prominently human leukocyte antigen (HLA) associations, have been shown to be enriched among responders (14–18) (Table 1). Given the role of HLA in antigen presentation and CD4 T cell activation, these data suggest a key requirement of CD4 T cells in alloantibody formation. However, alloimmunization may also occur independent of HLA type (19), suggesting that CD4 T cell–independent (TI) responses may also occur. A variety of HLA-independent polymorphisms have also been linked to responder status, although the mechanistic basis for many of these associations remains to be explored (14–17).

In addition to genetic factors, the underlying disease state of patients at the time of transfusion may influence the likelihood of RBC alloimmunization (Table 1). Unlike vaccination or infection, RBCs do not possess known adjuvant properties thought to be required for a robust adaptive immune response (20, 21). However, patients transfused during a febrile episode are more likely to develop alloantibodies (22), suggesting that inflammatory events associated with fever may prime the immune system and therefore serve as a surrogate for adjuvants that sensitize individuals to become alloimmunized. Infection itself may likewise influence alloimmunization following transfusion. However, infection can have a dichotomous impact on alloimmunization. Transfusion during a viral infection trends toward increased rates of alloimmunization, while patients with gram-negative sepsis have the opposite predilection (23), suggesting that not all forms of inflammation similarly impact the likelihood of RBC alloantibody formation.

In addition to more acute aspects of a patient’s status at the time of transfusion, chronic underlying disease conditions also appear to influence the likelihood of alloimmunization (Table 1). Patients with sickle cell disease (SCD), myelodysplastic syndrome, hereditary telangiectasia, systemic lupus erythematosus, and rheumatoid arthritis have been associated with alterations in immune activation and increased risk of alloimmunization (24, 25). In contrast, patients with conditions that may compromise immune function, such as bone marrow failure syndromes, acute myeloid or lymphoid leukemia, or chronic liver or kidney failure, or who are undergoing treatment for solid tumors or are on immunosuppressive drugs in general, are less likely to develop alloantibodies following RBC transfusion (24, 26–29). It should be noted that among patients with SCD, transfusion during complications associated with inflammation [acute chest syndrome (ACS) and vaso-occlusive crisis (VOC)] significantly increases the risk of alloimmunization, while transfusion during events not associated with systemic inflammation, such as stroke, is not associated with an increased alloimmunization rate (30). These results contribute to growing evidence that recipient inflammation at the time of transfusion, either through acute events, chronic inflammatory states, or a combination thereof, may prime the immune system for an alloimmune response. Such priming events, coupled with underlying genetic factors that may also influence baseline immune function, could collectively influence RBC alloimmunization risk.

In addition to the possible impact of patient inflammatory states on RBC alloimmunization, several studies have suggested that changes in immune organ function, most notably the spleen (as a result of injury, surgical splenectomy, or other causes of altered function), may alter alloimmunization risk. Splenectomized individuals fail to generate anti-RBC antibodies following xenotransfusion (31), and recent retrospective data on allogeneic RBC alloimmunization corroborate these earlier results (32). The pivotal role for the spleen in RBC alloimmunization may not be surprising when considering its role in responding to blood-borne antigens in general (33). However, these findings may appear to conflict with the apparent proclivity of patients with SCD, many of whom are considered to be functionally asplenic, to generate alloantibodies following transfusion. Recent studies suggest that most patients with SCD actually possess residual spleen function, which can significantly increase following hydroxyurea therapy, a common treatment modality in patients with this condition, or RBC transfusion (34–36). Also, many patients with SCD may have become primed to generate an RBC alloantibody response or have developed alloantibodies following an earlier transfusion (prior to splenic infarction, for example). This is especially important when considering that the initial timing of RBC alloantigen exposure responsible for alloimmune priming events or even the development of detectable alloantibodies in retrospective studies most often remains unknown (37). As a result, while certain disease states may alter spleen architecture or function, earlier priming events may have already occurred, and, if these initial exposures have not transpired, in most cases the spleen may possess sufficient capacity to still play a role in RBC alloimmunization. In the complete absence of a spleen, critical immune priming events required for RBC alloimmunization may therefore be less likely to occur (31, 32, 38–40). However, it is certainly possible that alternative immune pathways may be engaged in some settings, such as inflammatory states that accompany infection or ACS, that can bypass the requirement of the spleen for RBC alloimmunization.

### Donor Factors

In addition to the impact of patient variables, features of the RBC unit itself may also influence the likelihood of RBC alloimmunization. Pretransfusion refrigerated storage, for example, changes RBC morphology, metabolism, and overall posttransfusion recovery (41). While conflicting data certainly exist (42, 43), one study in particular suggested that storage may have a bimodal impact on RBC alloimmunization, with intermediate lengths of storage enhancing RBC alloimmunization and greater storage lengths reducing or enhancing alloimmunization (30). In contrast, RBC irradiation, despite possible generation of damage-associated molecular patterns (DAMPs), does not appear to alter alloimmunization rates (44). Other product modifications, such as leukoreduction, may actually reduce the probability of RBC alloimmunization (45).

As alloimmunization is a relatively rare event, studies examining patient and donor features that correlate with alloimmunization often understandably employ pooled alloimmunization data that represent alloantibody formation against a variety of alloantigens (46, 47). It is possible that some genetic and environmental variables may influence alloimmunization to distinct alloantigens differently. Such alloantigen-specific responses may account for apparent discrepancies regarding the impact of storage, genetic variables, recipient states at the time of transfusion, or other factors on alloimmunization risk given the distinct frequencies of alloantibodies present among the different patient populations represented in each study. As a result, future studies will be needed to dissect possible associations with specific patient and donor unit features that may place individuals at risk for alloimmunization toward individual alloantigens; given the frequency of alloimmunization, such an approach will likely require a coordinated effort between many centers.

## IN VITRO HUMAN STUDIES—THE RESPONDER–NONRESPONDER CONTINUUM

In addition to general features of population-level characteristics that appear to predict or at least correlate with RBC alloimmunization, recent studies have sought to define key features of general immune activity within patients that may correlate with responder or nonresponder status.

### General Evaluation of Immune Cell Numbers in Responders Versus Nonresponders

While most immune populations exhibit no difference between responders and nonresponders, several subsets of CD4 T cell memory are higher among responders versus nonresponders (48). Additional studies identified a reduced number of PD1^+^CXCR5^+^ circulating T follicular helper (Tfh) cells (including Tfh subsets) among alloimmunized compared with nonalloimmunized individuals (49). Further examination suggested that responders may also have lower expression of Fc gamma receptor type 1 (FcγR1) on classical and intermediate monocytes than nonresponders(50).

### Evaluation of Immune Cell Function in Responders and Nonresponders with a Focus on Heme

**Figure 2:**
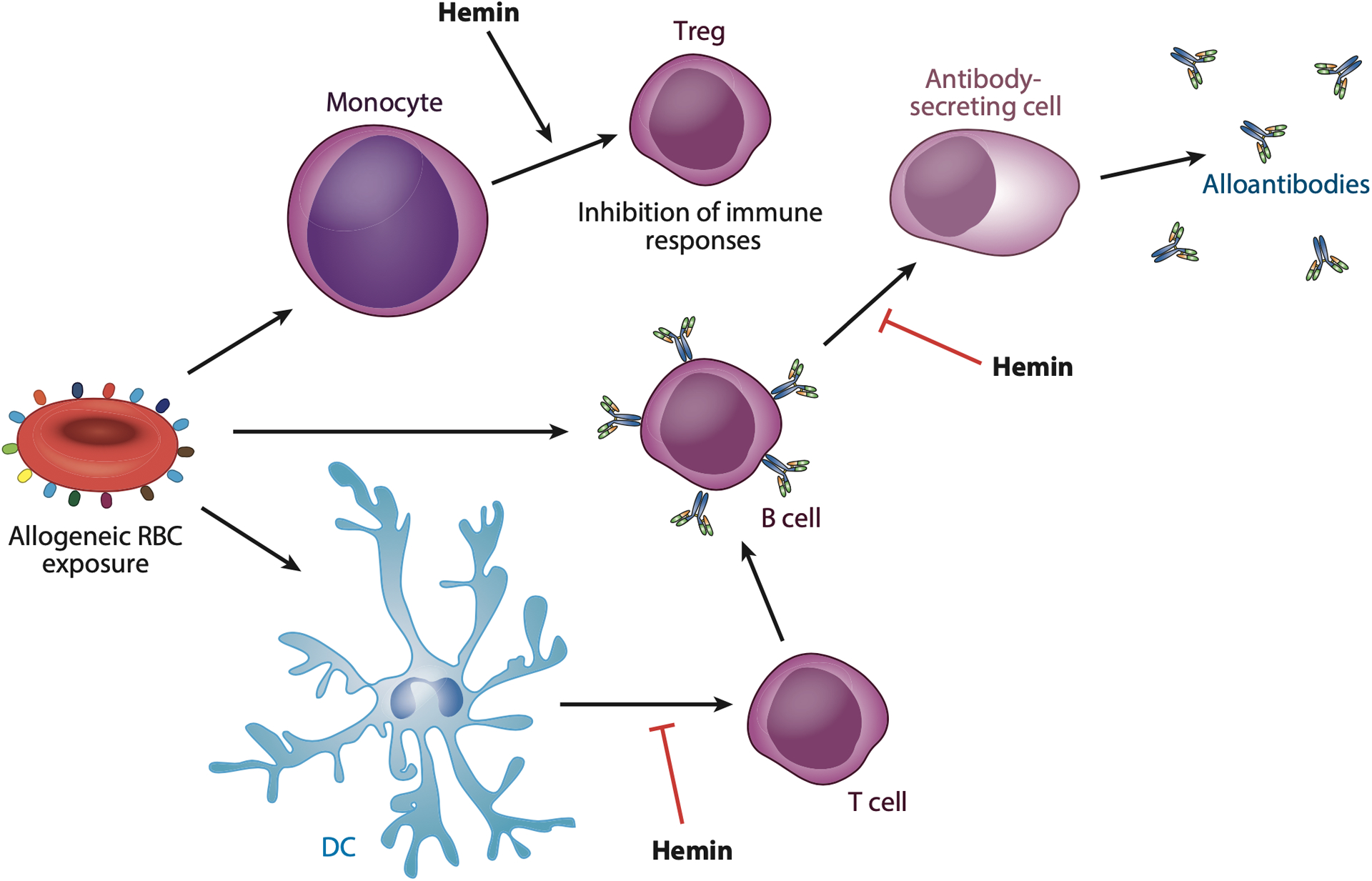
Hemin can regulate immune cell function in a responder- or nonresponder-specific manner. The apparent proclivity of some individuals to respond to allogeneic red blood cell (RBC) transfusion by generating alloantibodies, while others do not, could in part be influenced by common functional differences in immune cell behavior between responders and nonresponders. Hemin not only appears to impair the ability of monocytes and monocyte-derived dendritic cells (DCs) to activate CD4 T cells but also enhances the development of regulatory T cells (Tregs), which can further inhibit immune function. Hemin also decreases the ability of CD4 T cells to simulate B cells to become antibody-secreting cells. Elevated levels of hemin, especially during disease exacerbations in patients with sickle cell disease, may therefore suppress the ability of many immune cells involved in alloantibody production to facilitate RBC alloimmunization. In contrast, several studies have demonstrated that alloantibody responders are not as sensitive to hemin-induced inhibition of immune function, possibly leading to an increased likelihood that alloantibodies form following RBC transfusion in this setting.

While no difference in regulatory T cell (Treg) numbers between responders and nonresponders has been identified, Treg activity is more pronounced in nonresponders than responders. Free heme, which is often elevated during exacerbations of SCD for which transfusion is likely to occur (51), can enhance Treg function through a heme oxygenase 1 (HO-1) and CD16^+^ monocyte-dependent process (52). Tregs from responders are less sensitive to heme-induced enhancement of Treg function (52). Additional studies suggest that Tregs from patients with SCD in general may exhibit some differences in chemotaxis from controls, although differences between responders and nonresponders may be less apparent (53) (Figure 2).

Tfh cells, which can be critical in antibody generation, can also differ between responders and nonresponders. TIGIT^+^ Tfh cells, which are particularly effective at producing interleukin (IL)-21 and IL-4 and inducing CD38^+^ B cell differentiation (54), are more effective at inducing B cell differentiation into plasma cells in responders than nonresponders (54). CD4 T cell migration in response to CXCL12 may likewise be delineated on the basis of responder status (53). Hemin can modulate CD4 T cell activation and interferon gamma (IFNγ) production by altering the activity of monocyte-derived dendritic cells (DCs). Hemin appears to achieve this outcome by inhibiting NF-κB and CD83 upregulation through a Toll-like receptor 4 (TLR4)-dependent process (55). Differences in CD4 T cell responses specifically to Jk^b^ have also been noted between responders and nonresponders (56).

B cell function can also differ between responders and nonresponders. While B cell proliferation is not impacted by hemin, differentiation into CD38^+^ antibody-producing plasmablasts is inhibited by hemin through an HO-1-dependent process. B cells isolated from responders, in contrast, exhibit less sensitivity to hemin-induced inhibition of B cell differentiation (57). The frequency of regulatory B cells (Bregs), similar to Tregs, is not different between responders and nonresponders. However, Breg stimulation results in more IL-10 production and greater inhibition of monocyte activation in nonresponders than responders, suggesting that differences in Breg activity may also contribute to the responder–nonresponder continuum (58).

In addition to distinct features of key immune cells involved in alloantibody production, variation in innate immune signaling may exist between responders and nonresponders. Patients with SCD have higher levels of myxovirus resistance protein 1, a downstream product of type 1 interferon (IFNαβ) activation (59). Despite this, IFNαβ levels do not appear to segregate responders from nonresponders, although B cells do express more of the activation marker CD86 at baseline among responders than nonresponders (59). Hemin can activate a variety of IFNαβ responses, which may contribute to the endotheliopathy reported in patients with SCD (59, 60). Thus, while IFNαβ may not be a key differentiator of responder status, it could influence alloimmunization in responders, especially when transfusion occurs during acute events in which IFNαβ production is heightened (60).

Collectively, these data suggest that responders and nonresponders possess varying monocyte, DC, T cell, and B cell activities and that hemin may be an important regulator of these responses. Transfusion events that occur during increased hemolysis, where heme levels and IFNαβ are elevated, may therefore represent a situation in which the responder status of an individual may be more likely to manifest, given the differential response to hemin and perhaps other inflammatory mediators along responder and nonresponder lines. Heme can have toxic effects, including inducing lung injury and facilitating complement activation during disease exacerbations (51, 61,62). Use of hemoglobin (Hb) or heme scavengers, such as haptoglobin or hemopexin, or inhibitors that target the downstream inflammatory consequences of Hb and heme, while possibly capable of favorably altering SCD pathophysiology, may also impact the likelihood of alloimmunization (63).

### The Development and Use of Preclinical Models: A Complementary Strategy to Studying RBC Alloimmunization

When seeking to prevent RBC alloimmunization, the identification of initiating events is important, as these pathways are often central nodes in the early alloimmune response that magnify later immune events that are more difficult to target. However, as only ABO(H) and RhD alloantigens are prophylactically matched in the emergent setting, deliberate exposure of individuals to RBC alloantigens for the express purpose of defining early immune events that govern RBC alloimmunization clinically is unethical, as it places patients at risk for HTRs should an emergency transfusion be required in the future. Furthermore, as the per unit incidence of alloimmunization is quite low (7), prospective studies seeking to capture alloimmunization events in transfused patients are also not practical. Even when alloantibodies are detected, as full alloantigen typing of donor units is not routinely performed and alloantibodies are often examined only prior to the next transfusion (which can occur at variable times following the most recent transfusion), the implicated unit and therefore the precise timing of alloimmunization is rarely known. As a result, examination of de novo alloantibody formation following RBC transfusion in patients is very difficult, which has made it challenging to understand the key initiating immune events that occur at the time of RBC alloimmunization clinically. This contrasts with other clinically relevant immune responses, such as those that occur following transplantation, vaccination, or infectious disease, where exposure events can be more accurately determined and therefore key features of the developing immune response can be defined.

Given the challenges associated with capturing RBC alloimmunization in real time, most studies evaluating patients have understandably instead examined global changes in immune function at baseline, often years following the alloimmunization event. However, even if prospective studies could be conducted in patients, key features of immunity to alloantigens require trafficking of antigens from blood into tissues, where critical immune populations reside (33, 64); the architecture of these extravascular immune compartments, including the distinct location and communication between unique immune cells, is critical for effective adaptive immune responses to occur (33). As these immune cells and the events they govern are difficult to study in peripheral blood alone, it can be challenging to define key players that drive RBC alloimmunization solely on the basis of in vitro studies; this has contributed to challenges in developing targeted approaches designed to prevent RBC alloimmunization (7). Given these challenges, preclinical models have been developed to study key aspects of initiating immune factors and tissue-resident populations that govern alloimmunization. In so doing, these models serve as a complementary approach to clinical studies, with the goal of identifying key principles that guide immune responses to transfused RBCs across mammalian species.

Unlike transplantation, where transfer of skin, for example, between distinct strains of mice results in alloimmune-mediated rejection due to differences in major histocompatibility complex (MHC) alloantigens, transfusion of RBCs between distinct strains of mice largely fails to result in detectable alloantibody formation. As a result, while MHC alloantigens in the setting of murine skin transplantation were described more than 70 years ago, comparable models capable of studying RBC alloimmunization have not been similarly available despite the discovery of RBC alloimmunization long before allogeneic barriers to transplantation were described (65, 66). Despite these limitations, several approaches have been attempted to study the immune responses to transfused RBCs in general. Sheep RBC–induced immune responses in mice have been examined for decades. However, the sheep RBC immune response can be difficult to interpret when seeking to model allogeneic transfusion, as these cells are cleared within minutes of transfusion (mouse RBCs typically circulate for 55 days); sheep RBCs also likely express many foreign antigens, often of unknown density and composition, that may contribute to an enhanced immune response. Differences in sheep RBC CD47 interactions with mouse signal regulatory protein α may also contribute to increased immunogenicity (67). Rabbits do express RBC alloantigens that can result in alloantibody formation following transfusion between different strains (68). However, the genetic and immunological tools available in mice to elucidate immune mechanisms of alloimmunization have unfortunately not been similarly generated for rabbits, precluding the opportunity to leverage the wealth of information and tools available in mice that are needed to uncover fundamental principles of RBC alloantibody formation. These limitations have directly contributed to a lack of comparable mechanistic understanding regarding the immune events that dictate alloantibody formation following transfusion when compared with other types of adaptive immune responses.

To overcome limitations in animal models, a variety of murine systems have been recently employed in the study of RBC alloimmunization. Several of these models, including the membrane-bound hen egg lysozyme (mHEL) (which expresses the HEL antigen on many cell types, including RBCs) and the glycophorin A (GPA) transgenic systems, were primarily generated for other purposes but have since been repurposed for the study of RBC alloimmunization (69, 70). Additional models, including the HOD system, which expresses chimeric antigen HEL, ovalbumin, and human Duffy on RBCs, and the KEL system, which expresses an unmodified human KEL antigen, were generated with the express purpose of studying RBC alloimmunization (71, 72). Importantly, distinct transgenic founders have been characterized that produce different densities of KEL and have been named KEL^hi^, KEL, and KEL^lo^ to indicate their relative levels of KEL expression (72–75). Duffy and RhD models have also been described but for unclear reasons have rarely been used to study alloimmunization (76–78). Each model exhibits unique features that likely provide important insight into the consequences of exposure to distinct RBC alloantigens. For example, the HOD antigen leverages existing immunological tools and therefore has provided important insight into antigen-specific responses following RBC transfusion (21, 40, 71, 79, 80), while the GPA, KEL^lo^, and KEL^hi^ systems have facilitated studies defining factors that influence tolerance or immune responsiveness following RBC transfusion (73, 74, 81, 82). The KEL system in particular recapitulates key features of clinically relevant alloimmunization, including the induction of alloantibodies capable of causing RBC clearance, HDFN, and acute HTRs (74, 83–87).

Similar to other preclinical models, including experimental autoimmune encephalitis for multiple sclerosis, the K/BxN serum transfer model for rheumatoid arthritis, and the dextran sodium sulfate/T cell adoptive transfer model for inflammatory bowel disease/colitis, each alloantigen system is a model with the inherent limitations of any preclinical system (including lack of data regarding whether each system accurately reflects alloimmunization following exposure to GPA, Duffy, or KEL clinically given the ethical challenges of deliberately exposing individuals to these alloantigens for experimental purposes). However, these models do recapitulate key features of clinically relevant alloimmunization and therefore may serve as general models of RBC alloimmunization to KEL, Duffy, GPA, or possibly other RBC alloantigens altogether.

Unlike the responder–nonresponder continuum observed clinically, for the most part, recipient responses in these model systems are largely dictated by the alloantigen involved and the recipient inflammatory state at the time of transfusion. As murine studies most commonly utilize inbred mice, these results may therefore more closely model responders in that all recipients examined can generate an alloimmune response. However, the likelihood and magnitude of the response appears to be influenced by donor and recipient variables, providing insight into key factors that may shape RBC alloimmunization within the responder continuum.

### Recipient Variables That May Influence Alloimmunization

Using preclinical models, reductionist approaches can be applied to define the impact of clinical variables that have been associated with RBC alloimmunization. As noted earlier, RBCs lack a known adjuvant property. As a result, early preclinical studies focused on the impact of deliberate induction of recipient inflammation on RBC alloantibody formation. These studies first examined the impact of viral-like inflammation. Recipients first exposed to the nucleic acid poly I:C (PIC), which mimics viral-induced immune activation, followed by transfusion of mHEL RBCs, had significant anti-mHEL antibody formation, whereas no detectable antibodies formed following mHEL RBC transfusion in the absence of PIC (9). This effect was not limited to PIC or mHEL RBCs. Transfusion of GPA RBCs in recipients primed with CpG, which can mimic bacterially derived DNA-induced inflammation, likewise results in anti-GPA antibodies, suggesting that a variety of adjuvants may enhance the likelihood of alloantibody formation (88). PIC also enhances alloantibody formation following KEL or HOD RBC transfusion (74, 83, 86), although in contrast to mHEL or GPA RBC transfusion, HOD and KEL RBC transfusion can induce alloantibodies, albeit at a lower level, in nonprimed recipients (74, 83, 86, 89). However, not all pathogen-associated molecular patterns (PAMPs) enhance alloantibody formation. Preexposure of recipients to lipopolysaccharide has the opposite outcome, inhibiting alloimmunization following transfusion of mHEL or HOD RBCs (21, 90). These results suggest that transfusion itself can induce nonresponsiveness but that the alloantigen target and the inflammatory state of the recipient may dictate whether nonresponsiveness or alloimmunization occurs.

While patients with SCD have clearly been shown to have an increased rate of alloantibody production on a per unit basis, transfusion of KEL or HOD RBCs into SCD disease mice fails to result in any difference in alloantibody levels when compared with wild-type (WT) recipients (91). However, as noted above, rates of alloimmunization in patients with SCD are increased only following transfusion during VOC or ACS (30), a result that has not been formally assessed in a model system, suggesting that while baseline SCD does not alter alloimmunization rates, disease exacerbations may contribute to enhanced alloimmunization. In contrast to the SCD model, CpG priming of thalassemia mice actually lowers the alloimmune response to GPA RBC transfusion (92).

### Donor Factors That May Influence Alloimmunization

In addition to recipient factors that may influence RBC alloimmunization, variability in the donor product can likewise impact rates of RBC alloantibody formation. This is especially important when considering that transfused RBCs are a biologic and, as such, can vary considerably between distinct blood donors (including differences in antigen levels and sensitivity to storage) and have general variation in processing and storage practices that may have distinct consequences on the immunological outcome of RBC alloantigen exposure (93).

#### Blood storage.

Given the possible association of storage with RBC alloimmunization clinically (30), the impact of storage has likewise been explored in several preclinical models with variable outcomes. For example, transfusion of stored HOD RBCs results in enhanced anti-HOD antibody formation (94). Transfusion of stored HOD RBCs also causes a systemic cytokine response (89, 94), suggesting that RBC storage may facilitate alloimmunization by enhancing innate immune activation. RBCs from different mouse strains exhibit distinct storage characteristics, which appear to directly impact HOD RBC circulatory life span and the development of anti-HOD antibodies following transfusion (95). In an elegant series of studies, Steap3 was identified as a key player in regulating different donor storage characteristics, suggesting polymorphisms in Steap3 or similar regulators of RBC storage may impact RBC alloimmunization (96). However, the impact of Steap3 polymorphisms on RBC alloimmunization remains to be tested.

Although the mechanism(s) whereby storage enhances HOD RBC alloimmunization remains incompletely defined, artificial methods designed to age or induce accelerated clearance of RBCs, such as heat injury, phenylhydrazine treatment, or even coating with antibodies that accelerate RBC clearance, can likewise enhance alloantibody formation following HOD RBC transfusion (75, 97, 98). Similarly, removal of the antiphagocytic marker CD47 from RBCs enhances RBC removal and antibody formation in the absence of any storage, heat, chemical treatment, or passive antibody-mediated removal, suggesting that simply accelerating RBC removal in the absence of RBC damage (heat or chemical treatment) or antibody coating may facilitate alloimmunization (99, 100). However, it should be noted that storage does not uniformly result in enhanced alloantibody development; storage decreases antibody formation following KEL RBC transfusion (101). Intriguingly, simultaneous transfusion of nonstored (fresh) and stored HOD RBCs partially reverses the impact of storage on enhanced alloantibody formation (102). These results illustrate that storage can have varied outcomes that are contingent on the target antigen engaged and the other units transfused. Such variables may contribute to challenges ascertaining the impact storage clinically, where, as noted previously, alloantibody responses to distinct alloantigens are pooled and therefore collectively evaluated. In addition, possible exposure to blood products with heterogeneous ages when patients receive multiple units may influence the impact of RBC storage on alloimmunization.

#### Donor RBC antigen levels.

Neither mHEL nor GPA RBC transfusion results in alloantibody formation in the absence of prior PIC priming. Even more intriguing, recipients transfused with mHEL or GPA RBCs remain unresponsive to additional challenge with the same antigen following PIC priming (82). These results suggest that some antigens may possess the ability to induce antigen-specific tolerance. While the mechanisms responsible for the distinct outcomes following unique alloantigen exposure events remain incompletely understood, the density of the antigen on the RBC surface, which can vary significantly between different blood donors, may play some role. This has been tested for the KEL antigen, where transgenic founders that produce RBCs with distinct levels of KEL are available. Transfusion of KEL RBCs, which express a similar level of KEL as exists on most human RBCs (87), results in alloantibody formation. However, transfusion of KEL^lo^ or KEL^hi^ RBCs fails to result in immunoglobulin G (IgG) anti-KEL antibody development. Recipients transfused with KEL RBCs that have previously been exposed to KEL^lo^ or KEL^hi^ RBCs do not generate detectable anti-KEL alloantibodies, suggesting that similar to mHEL and GPA RBC exposure, prior exposure to KEL^lo^ or KEL^hi^ RBCs can render recipients unresponsive to KEL alloimmunization (73, 85). This lack of responsiveness appears to be antigen specific, as transfusion of these same recipients with HOD RBCs does result in detectable anti-HOD antibodies (73). These results suggest not only that donor unit antigen levels may influence the likelihood of alloimmunization but also that the timing and recipient conditions under which alloantigen exposure occurs may impact subsequent responsiveness to a particular antigen. In this way, nonresponder status may not always reflect recipient characteristics that globally influence immune function and therefore the likelihood of alloimmunization in general, but it may also reflect the historical exposure to an alloantigen in a given patient. Nonresponsiveness may therefore also exhibit some degree of alloantigen specificity. As a result, even if a patient responds to one alloantigen, and is considered to be a responder, the same patient may be a nonresponder for another alloantigen. However, it is important to note that the antigen density and timing of exposure that result in unresponsiveness may be antigen specific; additional studies are certainly needed to explore these possibilities.

## INNATE IMMUNE PATHWAYS AND CELLS THAT INITIATE RBC ALLOIMMUNIZATION

### Mechanisms of Inflammation and RBC Storage on Alloimmunization

While early preclinical studies explored the general impact of recipient inflammatory states and donor unit characteristics on RBC alloimmunization, the actual innate immune pathways and cells that govern alloimmunization remained relatively unexplored. Early efforts designed to define these pathways first examined key antigen-presenting cell populations that may phagocytose transfused RBCs and therefore possibly present RBC alloantigen peptides to CD4 T cells, thereby facilitating the CD4 T cell activation and subsequent help to alloantigen-specific B cells. These studies demonstrated that while a variety of cells can phagocytose transfused RBCs, recipient priming with PIC enhances DC RBC uptake in particular (103), suggesting a role for DCs in CD4 T cell activation following RBC transfusion.

**Figure 3:**
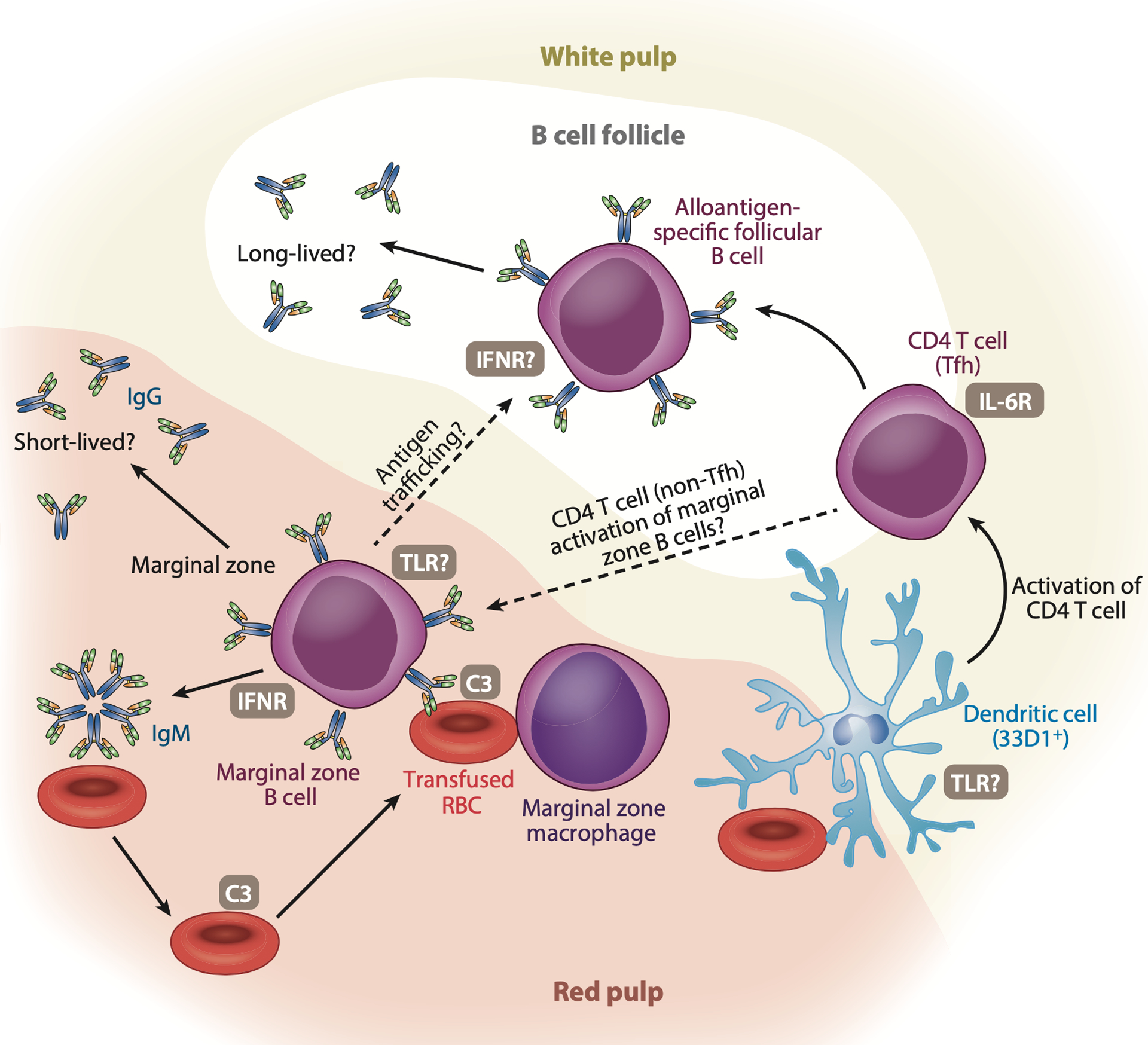
Cellular players and innate signaling pathways proposed to regulate red blood cell (RBC) alloimmunization. Allogeneic RBCs can be engaged by a variety of effector systems to induce alloantibodies. RBC removal by 33D1^+^ bridging channel dendritic cells (DCs) can result in the trafficking of antigens to the T cell zone in the white pulp, where direct activation of CD4 T cells, such as T follicular helper (Tfh) cells, can occur. RBCs can also engage marginal zone (MZ) B cells, which can result in the formation of immunoglobulin M (IgM), which can variably fix complement depending on the target antigen involved. Complement component 3 (C3)-coated RBCs may then engage distinct complement receptors to further promote alloimmunization. MZ B cells can generate IgG antibodies in the absence of CD4 T cells through a C3 and type 1 interferon receptor (IFNR)-dependent process. In contrast, CD4 T cell–dependent alloimmunization can likewise occur through a MZ B cell–dependent pathway, where the interleukin-6 receptor (IL-6R) on CD4 T cells and Toll-like receptors (TLRs) are involved. The distinct immune pathways engaged may have consequences on the persistence of alloantibodies following initial alloimmunization and the likelihood of an anamnestic alloimmune response should alloantigen reexposure occur.

Using RBC storage as a mechanism to enhance alloimmunization, additional studies demonstrated that splenic 33D1^+^ DCs in particular are potent activators of CD4 T cells ex vivo, and when these cells are deleted in vivo, CD4 T cell activation and IgG antibody formation following HOD RBC transfusion is prevented (21, 104, 105). While HOD RBC-induced alloimmunization occurs independent of the inflammasome or CD1d-mediated presentation of lipid mediators possibly generated during RBC storage (106, 107), HOD RBC-induced alloimmunization does rely on a MyD88-dependent pathway (108). Although the exact TLRs responsible for inducing MyD88-dependent signaling following transfusion remain to be determined, TLR4, which is responsive to heme (109), may play a key role. However, it should be noted that the recent discovery of TLR9 on the surface of RBCs raises the distinct possibility that TLR9-associated ligands on RBCs may drive TLR-mediated immune cell activation and subsequent alloantibody formation following RBC transfusion (110). The features of RBC storage that drive TLR-driven DC activation and eventual IgG production following HOD RBC transfusion remain unknown (Figure 3).

Transfusion of stored RBCs also results in marked elevation of IL-6, MCP1, and other cytokines (111), raising the possibility that the cytokine response to stored HOD RBC transfusion may contribute to alloantibody formation. Consistent with this possibility, transfusion of HOD RBCs into IL-6 receptor (IL6R) knockout (KO) mice results in a significantly blunted alloantibody response (112). Interestingly, while virtually no difference in serum cytokine response to HOD RBC transfusion was observed in IL6R KO mice, reductions in antigen-specific CXCR5^+^ PD1^+^ and CXCR5^+^ BCL6^+^ Tfh cells were observed, strongly suggesting that IL-6 regulates HOD RBC alloimmunization through a Tfh-dependent process (112). As MyD88 KO mice fail to generate a significant systemic cytokine response (including IL-6) in response to stored HOD RBC transfusion (108), MyD88 regulation of IL-6 production may reflect an important initial step in HOD alloimmunization. However, the possible role of 33D1^+^ DCs or other immune cells in sensing MyD88 signaling events and the source of IL-6 production remain to be determined.

Given the ability of PIC to increase IFNαβ (85), the possible role of IFNαβ on RBC alloimmunization has also been explored by transfusing recipients in which the common IFN receptor (IFNR), through which all IFNαβs signal, has been deleted (IFNR KO) (85). KEL^hi^ RBC transfusion of PIC-primed IFNR KO recipients fails to result in any detectable anti-KEL antibodies (85), demonstrating a key role of IFNαβ in this immune response. IFNR expression on B cells appears to be critical for this pathway (81), although DCs may certainly play a role in IFNαβ production and/or other aspects of KEL alloimmunization. This PIC-priming pathway appears to require the mitochondrial antiviral-signaling protein and interferon regulatory factors 3/7 (IRF3/7) pathway (85). Recipients can also be similarly sensitized to KEL^hi^ RBC-induced alloimmunization by actual influenza infection through the same IFNR- and IRF3/7-dependent pathway (113).

### RBC-Induced Alloimmunization in the Absence of Priming

While early studies focused on the ability of PIC exposure and RBC storage to enhance alloimmunization, it is important to note that HOD and KEL RBCs can induce alloantibody formation in the absence of known recipient inflammation or RBC storage (although KEL RBCs are more potent at inducing IgG antibody formation in this setting than HOD RBCs). This is especially intriguing when considering that beyond the possible role of nucleic acids bound to TLR9 outlined above (110), PAMPs and DAMPs, which are innate immune activators typically thought to be required for a productive adaptive immune response (108), are not know to be present in RBCs. The ability of RBCs to induce immune responses in the absence of known PAMPs or DAMPs is not unique to RBC transfusion, as treatment of patients with hemophilia with the factor VIII blood-clotting protein (which likewise does not possess known PAMPs or DAMPs) can also induce alloantibodies along a responder–nonresponder continuum (114, 115). In an effort to define early immune players that may trigger alloimmunization and therefore provide possible insight into how RBC alloantibody responses occur in the absence of a known adjuvant, the immune populations that engage intact RBCs shortly after transfusion have been explored.

Given clinical data demonstrating that the spleen can be a critical player in the development of RBC alloantibodies (31, 32), unique immune cells in the spleen may be the first responders in alloimmunization (38–40). The possible role of spleen-resident immune cells is even more apparent when considering that the spleen provides a unique setting in which transfused RBCs in blood can directly interact with immune constituents at sites where the red pulp (where red blood cells predominately reside) interfaces with the white pulp (where most immune cells, including key B cell populations that differentiate into antibody secreting cells, reside); this organization is relatively unique to the spleen and does not typically occur in most other secondary lymphoid organs (33). Consistent with this possibility, not only is the spleen required for RBC alloimmunization in preclinical models (38–40), but KEL and HOD RBCs localize to the marginal zone (MZ) of the spleen, a zone that resides at the interface of the red pulp and the white pulp (39, 40). Within the MZ, KEL and HOD RBCs colocalize in particular with MZ B cells, a distinct innate-like B cell population that can rapidly respond to blood-borne antigens (116). MZ B cells also exhibit a partially activated phenotype with a lower threshold for antibody generation, suggesting that these cells may be uniquely poised to respond to RBC alloantigens in the absence of a known adjuvant (116). Consistent with this, depletion or genetic deletion of MZ B cells can prevent or significantly reduce KEL or HOD RBC-induced IgM and IgG alloantibody formation (39, 40). While MZ B cells have been shown to be potent activators of CD4 T cells, MZ B cells do not directly activate CD4 T cells or traffic antigens to the B cell follicle in response to HOD RBC transfusion but instead appear to generate CD4 T cell–dependent (TD) IgG antibodies directly (40). In contrast to HOD alloimmunization, IgG anti-KEL antibody formation occurs in the complete absence of CD4 T cells (39, 83, 117). Thus, while MZ B cells are required for initiating responses to KEL and HOD RBCs, these antigens possess the intriguing ability to induce IgG antibody formation through TD and TI pathways (39, 40, 83, 117) (Figure 3).

MZ B cell–dependent IgM antibodies are TI following HOD or KEL RBC transfusion (39, 40, 117), suggesting that early MZ B cell–mediated IgM production may set the stage for downstream immune events that dictate the requirement of CD4 T cells for IgG antibody production. As IgM is an efficient complement activator in general and complement can be a potent enhancer of humoral immune responses (118), early IgM antibody binding to transfused RBCs may trigger complement activation that may then serve as a host-derived adjuvant of RBC alloimmunization. Differences in initial complement activation following IgM formation against KEL or HOD could therefore drive distinct downstream immune pathways, providing a possible explanation for the ability of KEL, but not HOD, to induce a TI IgG antibody response. Consistent with this, significantly more complement component 3 (C3) is indeed present on KEL RBCs when compared with HOD RBCs at the peak of the IgM antibody response, despite similar overall IgM antibody levels (119). In the absence of C3, IgG anti-KEL antibody formation becomes TD, demonstrating that C3 is required for TI IgG antibody formation (117). Interestingly, the immune response to KEL in the absence of C3 is enhanced when compared with WT recipients (117, 119, 120), standing in stark contrast to many other humoral immune responses that actually require C3 for IgG formation (121, 122). While this difference could reflect a complete reliance on CD4 T cells in the absence of C3, it could also result from the ability of C3 to remove the KEL antigen from RBCs during the ongoing immune response and therefore reduce antigen availability over time (119).

While a variety of complement receptors may facilitate C3-dependent, TI IgG anti-KEL antibody formation, the expression of high levels of complement receptor 2 (CD21) is a distinguishing feature of MZ B cells (116), suggesting that CD21 may drive this pathway. Consistent with this, CD21 on B cells is required for CD4 TI IgG antibody formation to KEL (117). Equally important, C3 may work in concert with IFNαβ to regulate alloantibody formation to KEL RBCs, as IFNR on B cells is required for anti-KEL antibody formation even in the absence of recipient priming with PIC (81). As MZ B cells and macrophages within the marginal sinus that support MZ B cell function are required for RBC alloimmunization in these models (123), these collective results suggest that IFNαβ and C3 may engage MZ B cells and other cells in the MZ to drive TI responses in the absence of a known adjuvant. In contrast, as IgM antibodies to HOD RBCs are not as efficient at fixing C3 (119), the need for storage to mount a strong IgG antibody response following HOD RBC transfusion may reflect the activation of storage-induced DAMPs that engage TLR pathways (108).

### Mechanisms of RBC-Induced Immune Unresponsiveness

The ability of transfused recipients to become unresponsive to additional alloantigen challenge was a somewhat unexpected finding in preclinical studies and suggested that RBC transfusion can actively modify immune function in recipients despite not inducing detectable alloantibodies (73, 75, 82). Given the central role of B cells and MZ B cells in particular in initially engaging transfused RBCs (39, 40, 124), B cells may represent the primary target of tolerogenic transfusion outcomes. The possible impact of antigen density itself on B cell signaling is intriguing when considering that in contrast to nearly all other signaling events propagated by receptor ligation, B cells must respond to an unknown antigen determinant sufficiently to initiate a signaling cascade that results in B cell activation, proliferation, and differentiation. Indeed, the B cell receptor (BCR) is much different than most host interactions with ligands that are largely invariant, even when considering engagement of microbial determinants, such as PAMPs or DAMPs. Even T cell receptors (TCRs), which can engage a broad range of foreign peptides, must do so in the context of MHC, which provides a much more predictable ligand; while peptide concentrations can differ, presentation to TCRs is ultimately dictated by the number of MHC molecules present. As a result, BCR signaling in naive B cells may be the only receptor system that develops direct ligand engagement abilities without interacting with its ligand. As a result, variations in the scaffolding, overall affinity, orientation, and other biochemical features of the antigen likely impact the outcome of this interaction. Given the diverse nature of RBC alloantigens (Figure 1), the distinct consequences of alloantigen exposure may reflect the unique impact of distinct alloantigens on BCR engagement, from differentiation into antibody secreting cells to immune tolerance.

While the unique features of B cell signaling raise the possibility that distinct antigen configurations may directly impact the consequence of B cell interactions, examination of antigen density or other antigen features on an immune response can be difficult. For example, chemically coupling a model antigen to an RBC surface can result in the formation of neoantigens at distinct attachment sites, making it difficult to separate the influence of responses to many different attachment-site epitopes from the model antigen itself (125). The KEL system has provided an opportunity to overcome some of these challenges and address the possibility that antigen density may in part influence B cell outcomes. Consistent with the possibility that transfusion-induced tolerance may influence B cells directly, B cells become uniquely unresponsive to KEL following KEL^lo^ RBC transfusion (73). However, whether KEL^lo^ RBC transfusion also impacts other immune cell populations, which in turn may impact alloimmunization, remains to be fully examined.

Exposure to an alloantigen in the absence of CD4 T cells may increase the likelihood that antigen-specific B cell tolerance occurs. Depletion of CD4 T cells or inhibition of CD40L in the setting of GPA transfusion can render recipients unresponsive to GPA even in the face of PIC priming (126). Unlike KEL RBC transfusion where removal of Tregs fails to alter KEL^lo^ RBC-induced tolerance or KEL RBC-driven alloimmunization in general (73), depletion of Tregs enhances alloantibody formation against GPA, while adoptive transfer of Tregs suppresses the anti-GPA response (88). Although Treg numbers may not predict anti-GPA responsiveness, the overall activity of Tregs does influence the likelihood and magnitude of the anti-GPA alloantibody response (127). These results suggest that while B cells may serve as a central node in translating the outcome of exposure to unique antigen densities, other key immune regulators, such as Tregs, can likewise influence overall immune responsiveness to RBC transfusion.

### Remote Prior Exposure Events Can Prime Recipient Alloimmunization

While exposure to PIC within hours of transfusion can sensitize recipients to become alloimmunized following transfusion, variability exists in alloimmune responses when inflammatory cues occur days to a week prior to transfusion (21, 85, 128). It is possible that distinct remote exposure events could induce increased numbers of antigen-specific memory cells independent of lingering innate inflammatory activation that then lowers the threshold for RBC alloimmunization following transfusion. Consistent with this, prior infection with a virus that harbors a sequence similar to HOD can facilitate the development of a memory T cell response that increases alloantibody formation following HOD RBC transfusion (80).

While exposure to microbes that mimic blood group antigens may directly facilitate subsequent alloimmunization, clinically, alloimmunization to one alloantigen in some patients can also directly increase the probability of subsequent alloimmunization (46, 47). In contrast to the TI response that KEL RBCs induce, PIC priming prior to KEL RBC transfusion boosts the KEL response through a TD process. Transfusion of previously PIC-and KEL-primed recipients with HOD and KEL double-positive RBCs results in an enhanced alloantibody response to the unrelated HOD antigen (83). While prior exposure to alloantibodies can inhibit or even enhance alloantibody formation (79, 84, 97, 129, 130), this priming does not rely on anti-KEL antibodies, as adoptive transfer of CD4 T cells from PIC-primed, KEL-transfused recipients alone recapitulated this outcome (83). These results suggest that prior alloimmune events to one alloantigen can directly facilitate alloimmunization to a completely distinct alloantigen following subsequent transfusion through a TD process. Such priming events may contribute to the underlying mechanism whereby some individuals have a higher probability of making additional alloantibodies following initial alloimmunization (47, 131). The ability of B cells to remove a portion of the cell membrane and associated cytoplasmic contents likely reflects trogocytosis (132), a process that has implications in the ability of B cells to acquire antigen from RBCs and receive CD4 T cell help following allogeneic RBC exposure.

These collective findings have clinical implications, not only with respect to possible targets that could be used to actively prevent RBC alloimmunization but also in uncovering key features of a recipient’s immune system that may aid in predicting whether an alloimmune response will occur. MZ B cells in particular possess a limited BCR repertoire (116), suggesting that differences in the precursor frequency of MZ B cells toward a given alloantigen, in addition to other key features of a recipient’s immune status, may in part influence the likelihood that an individual perceives and subsequently responds to RBC alloantigen exposure. However, additional studies are certainly needed. The mechanistic underpinnings of RBC alloimmunization are certainly more complex and nuanced than previously recognized.

## WHAT IS THE CONSEQUENCE OF RBC ALLOIMMUNIZATION?

As the consequences of incompatible transfusion have been beautifully reviewed elsewhere (133), we only briefly cover this topic here. HTRs are not the inevitable outcome of an incompatible RBC transfusion. However, methods to predict incompatible transfusion outcomes remain limited, especially in the acute setting. As a result, clinical strategies are designed to avoid HTRs by procuring compatible RBC units. Unfortunately, in heavily alloimmunized individuals, this approach may not be possible (134–136).

**Figure 4:**
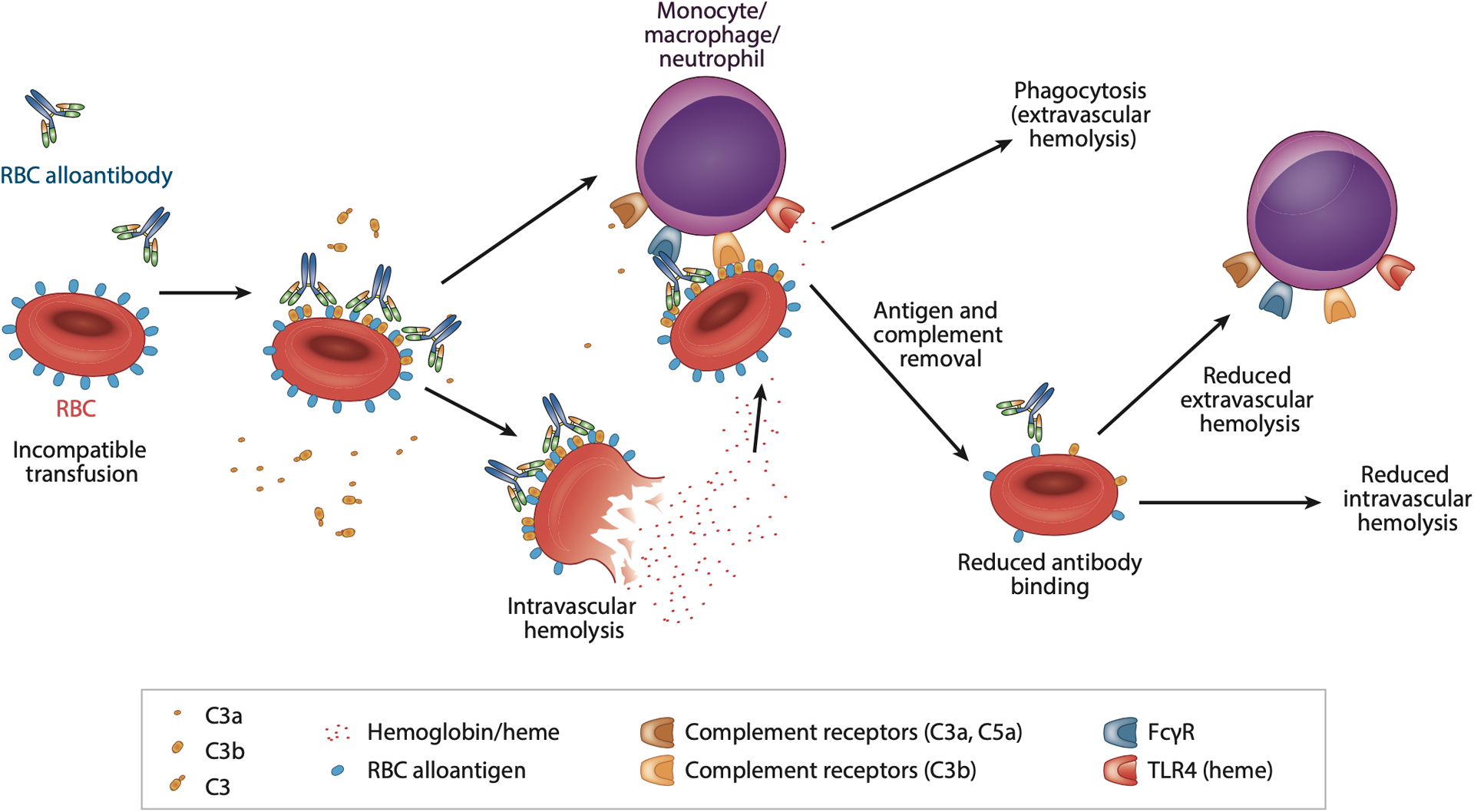
Alloantibody engagement of red blood cells (RBCs) following an incompatible transfusion can result in varied outcomes. Alloantibodies can induce complement activation, Fc gamma receptor (FcγR) ligation, or both, which can lead to extravascular and/or intravascular hemolysis. The antibody effector system involved in RBC removal also appears to facilitate antibody-induced loss of the target antigen. Loss of the target antigen lowers antibody binding, reducing the ability of antibody effector systems to facilitate extravascular and/or intravascular hemolysis and allowing transfused cells that have experienced antigen loss to persist in circulation despite the presence of the offending alloantibody. Complement split products generated following complement activation can further activate systemic immune responses, while heme released following a hemolytic transfusion reaction (HTR) can induce additional complement and immune cell activation through activation of innate immune receptors, including complement receptors and Toll-like receptor 4 (TLR4). Recipient variation in phagocytic function and/or complement levels may also influence each of these pathways. The consequences of incompatible RBC transfusion can therefore be quite variable depending on the alloantibodies and alloantigens involved, which can impact the extent of RBC clearance versus antigen loss, the activation of complement, and the release of free heme, all of which can influence the clinical sequelae of an incompatible transfusion. As a result of the varied consequences of antibody binding to RBCs, it can be difficult to predict the outcome of a given incompatible RBC transfusion a priori or devise common approaches to prevent or treat all HTRs should only incompatible RBCs be available.

Given the potentially deleterious consequences of an incompatible RBC transfusion, it is difficult to define mechanisms of immune-mediated RBC clearance clinically. Efforts to elucidate mechanisms whereby antibodies clear incompatible RBCs have therefore utilized the same preclinical models outlined above. Using these models, antibody-mediated GPA RBC clearance has been shown to rely on C3 or activating FcγRs (137), while anti-Duffy-mediated HOD RBC removal is entirely FcγR dependent (138). Anti-KEL antibodies likewise induce KEL RBC removal through an FcγR- and C3-dependent process, whereas anti-HEL antibodies can induce selective HEL antigen removal in the absence of noticeable RBC clearance through FcγRs or through a process independent of either FcγRs or C3 depending on whether mHEL or HOD RBCs are employed, respectively (84, 87, 139–141). Taken together, these results suggest that the types of antibody effector systems engaged can vary in part on the basis of the target antigen involved, although the type of antibody can certainly influence incompatible RBC transfusion outcomes (79) (Figure 4).

After the initial wave of KEL, HOD, or GPA RBC clearance following an incompatible transfusion, the rate of RBC removal can abruptly decrease (87, 138, 142). Antibody and antigen levels on transfused RBCs in each of these systems decrease in parallel with changes in clearance, suggesting that decreases in target antigens may reduce RBC sensitivity to antibody-mediated removal (75, 87, 138, 142, 143). Antigen removal following incompatible transfusion likely reflects complete loss of the target antigen, as opposed to antigen internalization or masking, as Western blot analysis fails to detect the antigen in this setting (84, 119, 141, 144, 145). These results suggest that when antibody levels fail to reach or fall below a threshold required for RBC removal, antibodies may simply persist on the RBC surface without impacting RBC survival.

Many variables, from the type of antibody and target antigen involved to the recipient immune status at the time of transfusion, likely contribute to the distinct outcomes observed following incompatible RBC transfusion. For example, antibody subclass and Fc glycosylation can dictate antibody activity (146). Additionally, the density, lateral mobility, and overall accessibility of target epitopes on a blood group antigen likely influence whether optimal C1q, FcγRs, both, or neither can be recruited by alloantibodies following RBC engagement (147). The overall function of phagocytic cells and/or complement levels in a transfusion recipient may also influence incompatible transfusion outcomes. The ability of antibodies to induce antigen loss in particular, which has also been observed clinically (145, 148), raises the possibility of designing strategies to selectively induce antigen loss independent of RBC removal as a mechanism of preventing antibody-induced HTRs. As incompatible platelet transfusion is common and does not result in significant morbidity or morality (149), antibody engagement of effector systems alone is not likely responsible for the adverse consequences of incompatible RBC transfusion. Instead, the rapid release of heme following acute hemolysis likely represents a significant driver of HTR pathophysiology, and therefore Hb and heme scavenging agents may be most effective at preventing or treating the deleterious consequences of an HTR. It should be noted that in the setting of delayed hemolytic transfusion reactions (DHTRs), anamnestic alloantibodies can result in an acceleration of allogeneic RBC removal over time (150, 151); heme-induced complement activation may also induce the hemolysis of the patient’s own RBCs, a particularly life-threatening process referred to as hyperhemolysis (152–154).

## CONCLUSIONS

In the last two decades, epidemiological, translational, and preclinical studies have provided new insight into the immune responses and overall consequences of RBC alloimmunization. These data suggest that alloimmunization is much more complex than previously appreciated, with unique immune pathways initiated following exposure to distinct alloantigens. RBC alloantigens are diverse. They differ significantly in form and function, in density, and in overall distribution, and these differences likely influence how sentinel immune factors engage these antigens and the overall consequence of alloantibody binding should an incompatible transfusion occur. The implementation of improved genotyping methods and databases that allow hospital transfusion services to more adequately match alloantigens prior to transfusion and be aware of historical alloantibodies that may have evanesced will be important when seeking to prevent alloimmunization and/or alloantigen reexposure in previously sensitized patients (155–158). Attempts to prevent Hb- and heme-induced immune activation are underway, as are studies seeking to define the utility of complement inhibition in preventing and treating DHTRs and associated hyperhemolysis (61, 62, 159, 160). Use of B cell depletion or plasma cell targeting approaches to prevent alloantibody formation in at-risk patients also has potential and awaits more formal evaluation (135, 161, 162). However, none of these approaches have fully eliminated the development and consequences of alloimmunization. As a result, additional studies are needed to enhance the precision of existing tools to further reduce the immune consequences of allogeneic RBC transfusion while carefully avoiding the unnecessary infectious risks that can occur following more global immune suppression. By doing so, a growing understanding regarding the development and consequences of RBC alloimmunization is possible and holds promise for the identification of more specific targets aimed at more effectively preventing and treating RBC alloimmunization and its attendant consequences.

## Figures and Tables

**Table 1:** Donor and recipient factors suggested to modify alloimmunization risk

Variable	Reduced alloimmunization	Increased alloimmunization
Recipient genetic factors	IL-10, TLR7, STAM, OX40L, IFNAR1, STAT4, IRF7, FCGR2, various HLAs	TNF, MALT1, TLR1, STAT1, TANK, IKK1, IL-2, ADRA1b, IL-6, IL-1B, CTLA4, various HLAs
Additional recipient factors	Gram negative infection	Viral infection
	Bone marrow failure	Autoimmunity
	Acute myeloid or lymphoid leukemia	Myelodysplastic syndrome
	Immunosuppressive drugs	Sickle cell disease^a^
	Splenectomy	
	Chronic liver or kidney failure	
RBC donor factors	Leukoreduction	RBC storage

a When transfusion occurs during specific complications, such as acute chest syndrome.

Abbreviations: HLA, human leukocyte antigen; RBC, red blood cell.
